# Adsorption in Action: Molecular Dynamics as a Tool to Study Adsorption at the Surface of Fine Plastic Particles in Aquatic Environments

**DOI:** 10.1021/acsomega.3c07488

**Published:** 2024-01-24

**Authors:** Piers A. Townsend

**Affiliations:** School of Applied Sciences, College of Health, Science and Society, University of the West of England (UWE), Bristol BS16 1QY, U.K.

## Abstract

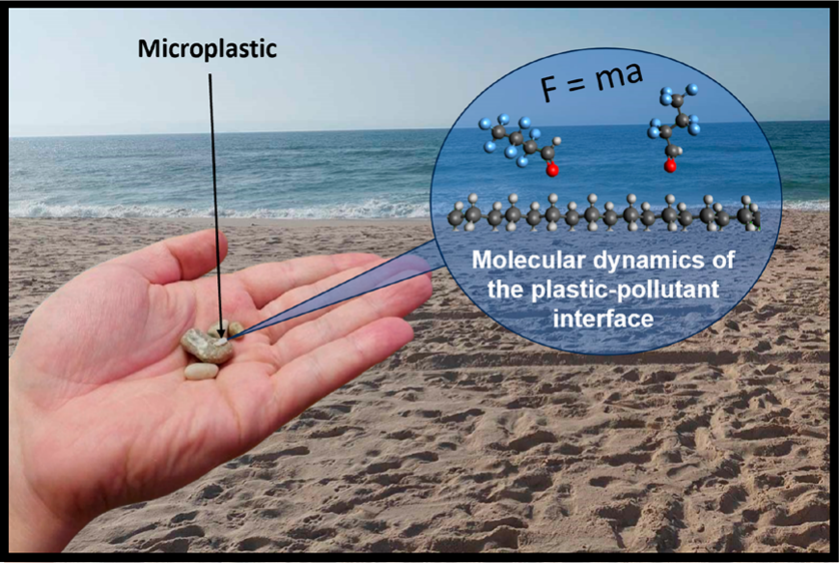

The presence of microscopic fine plastic particles (FPPs) in aquatic environments continues to be a societal issue of great concern. Further, the adsorption of pollutants and other macromolecules onto the surface of FPPs is a well-known phenomenon. To establish the adsorption behavior of pollutants and the adsorption capacity of different plastic materials, batch adsorption experiments are typically carried out, wherein known concentrations of a pollutant are added to a known amount of plastic. These experiments can be time-consuming and wasteful by design, and in this work, an alternative theoretical approach to considering the problem is reviewed. As a theoretical tool, molecular dynamics (MD) can be used to probe and understand adsorbent–adsorbate interactions at the molecular scale while also providing a powerful visual picture of how the adsorption process occurs. In recent years, numerous studies have emerged that used MD as a theoretical tool to study adsorption on FPPs, and in this work, these studies are presented and discussed across three main categories: (i) organic pollutants, (ii) inorganic pollutants, and (iii) biological macromolecules. Emphasis is placed on how MD-calculated interaction energies can align with experimental data from batch adsorption experiments, and particular consideration is given to how MD can complement existing approaches. This work demonstrates that MD can provide significant insight into the adsorption behavior of different pollutants, but modern approaches are lacking a generalized formula for theoretically predicting adsorption behavior. With more data, MD could be used as a robust, initial assessment tool for the prioritization of chemical pollutants in the context of the microplastisphere, meaning that less time-consuming and potentially wasteful experiments would need to be carried out. With additional refinement, modern simulations will facilitate an improved understanding of chemical adsorption in aquatic environments.

## 1.0 Introduction

It is now undeniable that pollution of the environment by fine plastic particles (FPPs) presents itself as one of the great global challenges facing modern society.^1−3^ Microplastics (MPs) are typically defined as small particles, generally <5 mm in size, made of common synthetic polymers such as polyethylene (PE), polypropylene (PP), polyethylene terephthalate (PET), and polystyrene (PS).^4^ MPs are now highly ubiquitous in nature and have been found across virtually all marine ecosystems.^5^ Coupled with their now widespread distribution, microplastics have been linked with a range of negative consequences such as the adsorption (and consequent release) of pollutants and their uptake by marine organisms.^6^ Nanoplastics (NPs) are now also a growing concern as MP particles can degrade even further into smaller FPPs. Nanometer-scale FPPs have been found in both aquatic and terrestrial environments, with a range of potentially unforeseen effects due to their small size.^7,8^ Due to the many unanswered questions that exist regarding the ecotoxicological risks associated with FPPs, this is a fact of significant concern.^9,10^

The sorption of organic and inorganic pollutants onto the surface of microplastics is known to occur due to their high surface-area-to-volume ratio and hydrophobic properties.^11^ Additionally, MPs have been shown to accumulate pollutants in concentrations much larger than the surrounding aqueous environment.^12^ These facts, coupled with the overwhelmingly large surface area potentially provided by the entirety of the so-called microplastisphere, indicate that developing a detailed understanding of sorption processes at plastic interfaces has never been more vital.^12^ Common organic pollutants that have been shown to adsorb onto microplastic surfaces include well-known environmental pollutants such as polyaromatic hydrocarbons (PAHs), polybrominated diphenyl ethers (PBDEs), and a wide range of pharmaceutical and personal care products (PPCPs).^13−15^ These molecules therefore provide the best examples by which to study the process of adsorption at FPP interfaces. Broadly speaking, sorption processes are controlled by the strength of the molecular interactions between the adsorbate (pollutant) and the adsorbent (FPP) at the atomic scale. Thus, utilizing methods that enable an atomistic (or atomic-scale) understanding of adsorbate–adsorbent interactions will play a pivotal role in our future understanding of how and why different plastic types could act as vectors for organic pollutants, and studies within this domain started to emerge in 2019.^16^ First, however, brief consideration must be given to how adsorption on FPPs is typically described and quantified.

### 1.1 Fine Plastic Particle Adsorption Capacities

To calculate the adsorption capacity of polymers that are commonly found as both MPs and NPs and, in turn, the potential ability of a given pollutant to adsorb onto its surface, sorption experiments are typically carried out with batch methods, wherein known concentrations of a pollutant are added to known weights of plastic particles.^17,18^ Adsorption capacities for a particular FPP are typically calculated according to eq 1:
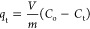
1where *q*_t_ is the adsorption capacity, *V* is the volume of a solution containing a potential pollutant, *m* is the mass of the sorbent, *C*_o_ is initial concentration of the pollutant, and *C*_t_ is the pollutant concentration at time *t*.^19^ Additionally, kinetic models are typically used to acquire an understanding of how quickly adsorption occurs and the possible mechanisms behind the process (e.g., pseudo-first-order (PFO) and pseudo-second-order (PSO)).^20^ Once the time taken to reach equilibrium has been established (which involves ascertaining when negligible changes in *q*_t_ occur), isotherm experiments can be conducted to acquire further mechanistic insight into a given adsorption process.^21^ Although batch methods are the traditional approach to studying adsorption (and calculating adsorption capacities), these experiments are often time-consuming and can be wasteful in their inherent design; in line with the 12 principles of green chemistry, the purchasing of pollutants should ultimately be minimized.^22^ This poses the following question: does there currently exist a less wasteful, more sustainable approach that could be utilized to understand adsorption and predict plastic–pollutant interactions?

### 1.2 Molecular Dynamics as an Alternative Approach

**Figure 1:**
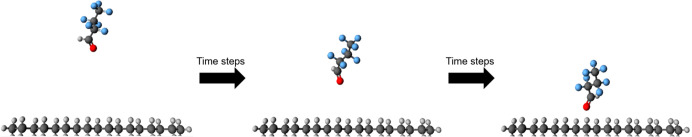
MD can be used to observe structural changes as a system evolves through time. Here, over numerous timesteps, a small PFAS (per- and polyfluoroalkyl substance) molecule can be seen approaching a hypothetical polyethylene surface.

At present, computational chemistry and molecular simulations have not been widely utilized to assess adsorption processes at FPP interfaces, despite offering a more sustainable approach, coupled with an unparalleled atomic-scale understanding of the structural changes and energetics of adsorbate–adsorbent interactions.^23,24^ These methods can be relatively low-cost and could potentially reduce the need for wasteful, unsustainable experimental approaches. In recent years, molecular dynamics (MD), a well-known theoretical approach for describing the physical movements of atoms and molecules, has been used to examine the adsorption of organic pollutants, inorganic pollutants, and other macromolecules both (i) on the surface of environmentally relevant polymers and (ii) at the polymer–water interface. MD involves predicting how the movement of each atom in a molecular system will change over time, wherein a physical model describing interatomic and intermolecular interactions is utilized. Once atomic positions have been initially specified, users can calculate the forces being exerted upon each atom (arising from the presence of other atoms in the simulation), allowing one to observe structural changes over a given timescale that can be chosen by the user (see Figure 1). In addition and vitally, MD simulations can provide the user with insight into the specific types of adsorbent–adsorbate interactions that are present, providing a highly detailed, fundamental understanding of the adsorption process. For example, there is often a delicate balance between short-range interactions (such as van der Waals interactions) and long-range interactions (such as electrostatic forces) that can be observed and calculated with MD simulations. Further, hydrophilic and hydrophobic interactions can play key roles in the adsorption process, both of which can also be observed by visualizing the output of MD simulations.^25^ For the sake of those unacquainted with MD, a number of key parameters are typically chosen for an MD simulation: these include temperature (controlled by a so-called thermostat), the force field (which provides the mathematical framework for energy calculations), the simulation timescale, and the thermodynamic ensemble.^26^ As of now, only a handful of publications have utilized MD in the context of microplastic adsorption processes, and these studies will be highlighted and discussed here in this work. First, however, a gentle introduction to molecular dynamics is presented for those unacquainted with the approach.

## 2.0 Molecular Dynamics: Molecules in Motion

The history of MD is vast and wide-ranging. In the late 1950s, the first MD simulations were carried out on a simple gas consisting of nondescript hard-sphere particles, and in the 1970s, the first MD simulation was performed on a protein, paving the way for a successful future for MD simulations across a range of disciplines.^27,28^ One of the largest drivers for the continued and popular use of MD simulations comes from the significant increase in the availability of computational resources in recent years; computers are growing increasingly more powerful, and locally run MD simulations with modest hardware are now readily possible due to modern graphics processing units (GPUs).^23^ However, for the largest-scale MD simulations, high-performance computing networks are still required. Despite this, the future is looking rather exciting for the field, with ab initio MD looking to play a pivotal role moving forward.^29^ MD software packages are also becoming easier than ever to use, allowing the method to be used by experts and non-experts alike; however, it must be noted that although MD simulations are easy to run in principle, significant expertise is still required to design accurate, well-founded simulations that can be meaningfully related to empirically observed phenomena. MD simulations provide a necessary bridge between macroscopic observations from a laboratory and the three-dimensional atomic-scale interactions that we cannot conventionally visualize. In essence, MD creates a powerful “molecular movie” of a chosen timescale (e.g., nanoseconds), where we can visually examine the ways in which assemblies of molecules interact with each other. MD simulations have previously been used to study a wide range of systems, ranging from large biomolecules to polymer composites that might be found in the marine environment.^30,31^ A range of biological processes have been studied and better understood with the use of MD; for example, methods such as DNA/RNA folding,^32^ protein folding,^33^ and enzyme catalysis.^34^ However, MD also has significant uses in areas such as materials science and toxicology. For example, MD can be used to better understand commercial polymers (and their interactions with pollutants), almost all of which account for the main FPP polymer types found in aquatic environments. MD has effectively been applied to a range of problems in polymeric materials such as the structure of polymer interfaces,^35^ polymeric membranes,^36^ polymer rheology,^37^ and diffusion phenomena.^38^ In addition to acquiring a better understanding of conformational and structural changes, MD can also be used to calculate thermodynamic properties such as free energies and equilibrium binding affinities.^39^

As discussed in the previous section, the basic premise of MD is actually quite simple. However, to understand its inner workings, some basic principles must be explored. To become an experienced practitioner in MD, there are many theoretical details that must be mastered, along with many practical choices that must be made when designing a simulation. Although not everything can be covered here in this Review, a few of the most important considerations will be discussed. For a more in-depth foray into MD, please see the excellent text from Binder and co-workers.^26^

### 2.1 Force Fields in Molecular Dynamics

**Figure 2:**
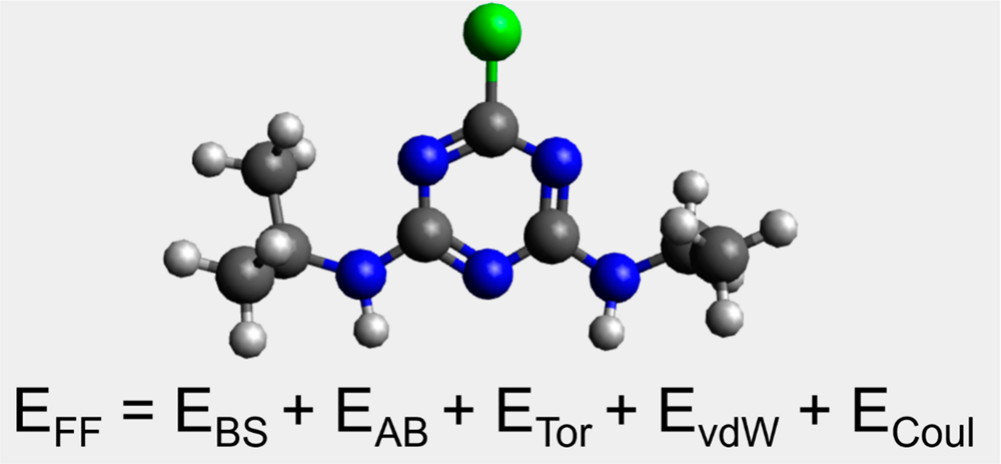
Force fields provide a mathematical description of inter- and intramolecular interactions.

At its core, MD simulations are solving Newton’s classical equations of motion for a molecular system:
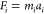
2where each atom *i* is part of a system of *N* interacting particles. In this equation, *m_i_* represents the mass of each atom in the simulation, *a_i_* is the acceleration of each atom, and *F_i_* is the force being exerted upon the *i*th atom due to the presence of other atoms in the simulation.^23^ Typically, the forces are then calculated through the use of a potential energy function:
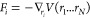
3where ∇ represents the Laplacian operator, and the overall term describes the gradient of the potential energy function with respect to the displacement of each atom. The potential energy function, *V*, tells the MD algorithm how each atom in the simulation interacts with everything else. This function is of the utmost importance in MD simulations; to obtain an accurate picture of the microscopic, atomic-scale behavior that arises due to classical Newtonian mechanics, a mathematical description of the interparticle interactions is required (see Figure 2). These interactions can typically be broken down into two types: (i) nonbonded interactions, wherein the atoms involved are not linked via covalent bonds, and (ii) bonded interactions, wherein the atoms would typically be covalently bonded to one another.^40^ For example, one of the simplest potentials is a simple pairwise interaction between atoms:
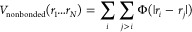
4where Φ represents a particular functional form to describe the potential between two atoms, and |*r_i_* – *r_j_*| represents the distance between atoms *i* and *j*. One of the best understood and most commonly used nonbonding potentials is the Lennard-Jones (LJ) potential:
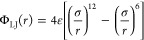
5where ε is the well depth, and σ is the van de Waals radius or, alternatively, the distance at which the particle–particle potential energy is zero.^41^ Then, for a system containing numerous atoms, the total potential can be calculated according to eq 4. If charges are present (e.g., a negatively charged carboxyl group is known to be present in some polymers), a Coulomb potential can also be included, which should be reminiscent of the all-familiar Coulomb’s law:
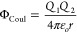
6where *Q*_1_ and *Q*_2_ represent the charges, *r* is the distance between the two charges, and ε_o_ is the permittivity of vacuum. A variety of functional forms exist for bonded interactions, for example, a two-atom bond could be modeled as a spring through means of a harmonic potential:
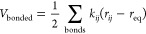
7where **k*_ij_* is the force constant, *r_ij_* is the bond length away from equilibrium, and *r*_eq_ is the equilibrium bond length, described mathematically as the minimum value of the harmonic potential. Now, vitally, the chosen force field in an MD simulation describes all of the functional forms of the bonded and nonbonded potentials and, therefore, how each atom interacts with another.^42^ Energy sits at the heart of molecular dynamics, and it is the job of the force field to, as accurately as possible, calculate the energy. It must be noted that force fields are inherently approximate, and they are typically created through the fitting of quantum mechanical data or by directly using experimental data in their parametrization.^43^ The force field has a significant effect on the overall calculated energy and can therefore significantly affect the quality of the results obtained. Numerous force fields exist in the literature, some of which have been designed very specifically for a particular use (e.g., PLAFF3 for polylactic acid, a sustainable compostable polymer),^44^ while others have been designed for broader usage. Commonly used force fields include CHARMM,^45^ AMBER,^46^ OPLS,^47^ UFF,^48^ and COMPASS,^49^ many of which have a whole host of variants and improvements relative to the original force field (e.g., OPLS3e).^50^ Choosing a force field is not a trivial matter, and it is one of the many choices that must be made prior to a simulation. For simulating adsorbent–adsorbate interactions, there is no clear answer on the best force field to use.^51^ Many commonly used force fields are often designed to reproduce experimental polymer properties (e.g., density) at specific sets of conditions, so unless there exists a force field built specifically for your polymer of interest, it is good practice to ensure that your key results are reproducible with different commonly employed force fields. Another important choice is the timescale and timestep in an MD simulation, and these are discussed in the next section.

### 2.2 The Importance of Timescale in Molecular Dynamics

As highlighted previously, MD algorithms involve solving Newton’s equations of motion, and one of the most commonly used approaches is the Verlet algorithm, of which many types and variants exist (e.g., the Leapfrog variant).^52^ The Verlet algorithm is a numerical approach to integrating the equations of motion and is responsible for acquiring a picture of where each atom will move over a given timestep. Put in another way, the Verlet algorithm enables the user to observe changes in the Cartesian coordinates of each atom across each simulation timestep. The mathematical definition of the Verlet algorithm is beyond the scope of this Review; however, the importance of the chosen timestep in Verlet integration must be discussed. Within the Verlet method, time must be discretized into intervals with each interval consisting of length Δ*t* (this is carried out to ensure numerical stability of the algorithm). Thus, the creator of an MD simulation must specify a timestep (Δ*t*) to use in their simulation. Typically, smaller timesteps (e.g., Δ*t* = 1 fs) will provide a more accurate picture of the system’s behavior, but this must be balanced with the computational resources available to the user.^53^ A common and typical timestep used in MD simulation is Δ*t* = 1 fs, and the timescale of the simulation is then determined by the sum of the number of timesteps used in the whole simulation. For example, if a simulation uses a timestep of 1 fs and involves a million iterations of the Verlet algorithm, a timescale of 1 ns will be represented across the whole simulation. It then becomes clear that larger timesteps will permit larger timescales to be accessed in the simulation, but unfortunately, accuracy and stability can be affected greatly by larger values of Δ*t*.^54^ In the case of adsorption processes, it has been previously noted that due to the timescale in which adsorption can occur (microseconds to hours), it is not always adequate to carry out simulations at nanosecond timescales.^55,56^ Choosing an appropriate timescale is a complex problem to which a whole text could and has been dedicated to, and its complexity must not be ignored by the non-expert; it is vital to ensure that a suitable timestep is chosen. Along with other parameters that must be chosen, such as the choice of the thermodynamic ensemble, the force field, and certain thermodynamic variables (e.g., temperature and pressure), MD simulations become a nontrivial exercise to perform. A few key factors are discussed in the next sections.

### 2.3 Choosing an Appropriate Thermodynamic Ensemble

As part of running an MD simulation, a thermodynamic ensemble must be selected to sample over the duration of the simulation. In their simplest and most intuitive form, MD simulations sample the NVE ensemble, otherwise known as the microcanonical ensemble.^57^ This involves keeping the number of particles (*N*, typically atoms in this context), the volume (*V*), and the total energy (*E*) constant in the simulation box. However, depending on the system under study and the problem at hand, other ensembles exist that can be sampled. For example, particle motion can be coupled to a figurative heat bath by using a thermostat, whereby the kinetic energy is maintained along with the temperature (the NVT or canonical ensemble).^58^ Alternatively, if the size of the simulation box is allowed to change, constant pressure can be maintained (resulting in the NPT ensemble), which represents the ensemble that can best be compared to real-world experimental conditions (where the pressure and temperature are kept constant in the laboratory). Choosing an ensemble is often an important choice to make, and the limitations of each must be considered, e.g., finite size effects can be present when the simulation box is allowed to change size.^59^ To illustrate the use of different thermodynamic ensembles from a practical perspective, a few different situations can be highlighted and linked to real-world applications. For example, if one was simulating a gas in an isolated container, where energy and mass transfer cannot take place between the system and its surrounding environment, then the NVE ensemble might be an optimal choice due to its operation with a constant number of particles (*N*) and constant energy (*E*). Alternatively, for biological simulations where very temperature-sensitive processes are being examined (e.g., protein dynamics), the NVT ensemble is often used, such that a constant temperature is maintained in the simulation box.^60,61^ By using the NVT ensemble, the effect of different temperatures on processes such as protein folding can be directly studied and visualized. Finally, if a process such as a phase transition is being simulated (e.g., the transition between two crystal structures), wherein the density of the material itself could change, the NPT ensemble should be utilized such that volume changes are allowed to take place.^62^ For modeling the interaction between FPPs and other molecules, the NPT/NVT ensembles would be strong choices due to their close link with standard experimental conditions. For example, batch adsorption experiments are typically carried out in the laboratory at a constant temperature (*T*) and pressure (*P*), meaning that the NPT ensemble is an obvious choice for this type of simulation. Along with the ensemble itself, choosing an appropriate temperature and pressure is also vital in the design of simulations that align with experimental data. For example, if adsorption experiments are carried out at 23 °C, it is vital that your MD simulations are representative of the same conditions.

### 2.4 Choosing an Appropriate System Size

**Figure 3:**
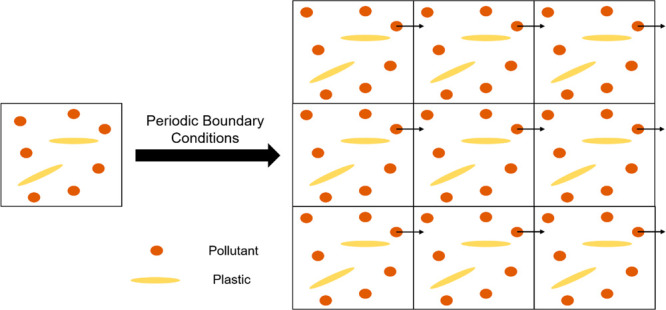
Graphical depiction of how periodic boundary conditions (PBCs) can be applied to an MD simulation. The arrows show pollutant molecules leaving from one side of the simulation box and entering from the opposite side.

Choosing the system size is a nontrivial choice due to a range of factors, including (i) computational cost and (ii) the existence of finite size effects.^59^ For example, in the case of adsorption onto the surface of an FPP, how does one ascertain the degree of polymerization (DoP) to use in the simulation? If smaller systems are chosen to avoid issues with computational cost, finite size effects can arise, leading to potential issues with calculation accuracies. However, the careful and considered usage of periodic boundary conditions (PBCs) can be employed to circumnavigate these issues. PBCs are commonly employed in MD simulations and involve “tricking” the simulation to behave as if it was infinite in size.^63^ The need for PBCs is best considered with bulk water; if half a gram of water was simulated, which at the macroscopic scale is a very small amount, ∼1.5 × 10^22^ molecules would be present in the simulation. This number is computationally intractable, leaving the question, how can one simulate bulk water? With PBCs, the simulation box is considered as a periodic unit cell, in which particles are free to move in the original simulation box. However, when an atom passes the boundary of the simulation box, it reappears on the other side of the simulation box. Essentially, the simulation box is surrounded with an infinite number of identical periodic images (see Figure 3). For finite size effects to be avoided, the cutoff distance (*r*_cut_) at which two atoms stop interacting must always be less than half the width of the simulation box, and this is typically known as the minimum image convention.^64^ If the cutoff radius is set larger than half the width of the simulation box, each atom could interact with multiple versions of “itself”, leading to significant inaccuracies in the energy calculations. In the context of FPPs, it has previously been shown that finite size effects can directly influence the dynamics of plastic materials, potentially having significant consequences on the understanding of atomic-scale phenomena.^65^ If the system size is likely to present a problem, coarse-graining offers an alternative approach for modeling large systems.^66^ These methods aim to reduce the computational complexity of a simulation by (i) reducing the number of degrees of freedom and (ii) removing fine interaction details, such as explicit atom–atom interactions. Additionally, they permit longer timescales to be accessed due to this reduction in complexity.^66^ For example, if a plastic polymer were to be simulated using coarse-grained MD, large, multi-atom areas of the polymer could be modeled as a single interactive entity, thereby reducing the number of dimensions that would need to be considered in the equations that describe the adsorbent–adsorbate interactions. With the drastic increase in FPPs and chemical pollutants in aquatic environments, it is vital that we develop an accurate picture of the atomic-scale adsorption processes occurring at the interface of FPPs and their surrounding environment (e.g. water). In the past few years, MD simulations have been used to great effect in this context, and the next section provides an overview of how MD simulations are elucidating molecular-scale adsorption processes at FPP interfaces.

## 3.0 The Application of Molecular Dynamics to FPP Adsorption

As of yet and broadly speaking, the application of MD to FPP adsorption falls into three categories: the interaction between FPPs and (i) organic pollutants, (ii) inorganic pollutants, and (iii) biological macromolecules, such as proteins and cell membranes.

### 3.1 Adsorption of Organic Pollutants to FPPs

**Figure 4:**
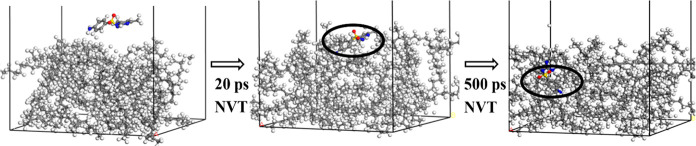
Adsorption of sulfamethazine onto the surface of a model PS particle over 500 ps of simulation. Reprinted with permission from ref (69). Copyright 2019 Elsevier.

It is widely accepted that organic pollutants can adsorb to the surface of FPPs in the marine environment, and understanding the interactions that occur at the plastic–pollutant interface is vital.^67^ At present, studies that examine the adsorption of organic pollutants on FPPs using MD simulations can be separated into two types: (i) those that use simulations in concert with experimental adsorption experiments and (ii) those that examine adsorption from an entirely theoretical perspective using MD and other computational methods. This section explores both types of studies, starting with those that use a combined approach. Antibiotics play a key role in society, and it has previously been shown that these compounds can adsorb onto the surface of FPP particles, resulting in the potential long-range transport of these chemicals.^68^ In 2019, Wang et al. published a combined experimental–theoretical approach in which the adsorption of antimicrobial sulfamethazine was studied using six different polymer types (PE, PET, PP, PS, PVC (polyvinyl chloride), and PA (polyamide)).^69^ They performed batch adsorption experiments where 20 mg of microplastic was added to a range of predefined concentrations of sulfamethazine. Additionally, adsorption isotherms were generated, and the effects of the pH and salinity were also considered. In concert with these experiments, MD simulations were carried out to predict the interaction energies when sulfamethazine adsorbed onto the six polymer types described above. Their simulations included polymer chains (with differing degrees of polymerization), a single molecule of sulfamethazine, and a so-called vacuum layer separating the polymer and sulfamethazine. In MD simulations, the vacuum layer is free space in the simulation box that permits the study of interfacial processes and attempts to prevent and minimize undesirable (and nonphysical) interactions from periodic boundary conditions.^70^ This study did not include any form of explicit solvent, such as water, which is a significant weakness in terms of simulating aquatic environments. They used the COMPASS force field in the NVT ensemble with a simulation time of 0.5 ns and a timestep of 1 fs. Their results showed that in the first stage of the simulation (0–20 ps), the adsorption of sulfamethazine was observed, followed by the diffusion of sulfamethazine into the polymer chains (20–500 ps, see Figure 4). Additionally, the interaction energy was calculated according to eq 8:
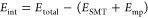
8where *E*_int_ is the interaction energy (kcal/mol), *E*_total_ is the energy of the model microplastic–sulfamethazine system, *E*_SMT_ is the energy of sulfamethazine, and *E*_mp_ is the energy of the polymer chain. *E*_int_ describes the strength of the interaction between the polymer and the pollutant, with more negative *E*_int_ values being associated with stronger adsorption on the FPP surface. It was shown that PA (−41.3 kcal/mol) and PET (−40.9 kcal/mol) polymer chains interacted most strongly with sulfamethazine, while PP (−12.1 kcal/mol) showed the weakest interaction among the polymer types included in the study. Interestingly and most importantly, the MD-calculated interaction energies were in alignment with the results obtained from the experimental isotherms. This is reassuring; thinking simply, it is intuitive to assume that more negative interaction energies will correlate with greater amounts of pollutant adsorbed experimentally, and to the best of the author’s knowledge, this study was the first to demonstrate a relationship between MD-derived interaction energies and FPP adsorption capacities. This study indicates that the trend observed in experimental adsorption studies can be effectively replicated by using computational MD approaches. Two years later in 2021, Chen et al. used a combination of MD and batch adsorption methods to study the adsorption of three antibiotics, namely tetracycline hydrochloride (TC), chlortetracycline hydrochloride (CTC), and oxytetracycline hydrochloride (OTC), onto the surface of polyethylene microplastics.^71^ Similar batch adsorption experiments to Wang et al. were conducted, but for their MD simulations, Chen et al. utilized the COMPASS force field in Materials Studio and modeled their FPP as a polyethylene chain consisting of 300 monomers of ethylene. Their simulations were performed over a short 0.3 ns timescale (1 fs timestep) and used a temperature of 298 K in the NVT ensemble. Their batch adsorption experiments showed that OTC had a higher (64.4 μg/g) adsorption capacity than CTC (63.4 μg/g), and in turn, CTC had a higher capacity than TC (53.5 μg/g). The effect of pH was also examined, and it was shown that adsorption was enhanced at a pH value of 6 before decreasing at higher pH values. Their MD results showed that CTC (−50.0 kcal/mol) was more strongly binding than OTC (−47.8 kcal/mol) by around 2.2 kcal/mol, while TC (−36.68 kcal/mol) had the weakest binding energy. Although the exact trend in adsorption capacity was not captured by the MD results, the adsorption capacities of OTC and CTC differed by only 1 μg/g; this could be indicative of many other factors, such as error present in the experiment and/or simulation. For example, choosing an appropriate force field is a nontrivial matter, and although COMPASS is known to perform well for soft matter simulations, in 2016, COMPASS was updated to COMPASS II, which included better parameters for common polymers and drug-like molecules found in popular databases.^72^ It is in this type of situation where force field validation would be of particular use, but no evidence of validation appears to be present in the study.^73^ Despite this, it is encouraging to see that even without robust testing of different simulation parameters, interaction energies calculated with MD can still reasonably align with experimentally calculated adsorption capacities. Similar to antibiotics, pesticides have also been shown to adsorb to FPPs,^74,75^ and in the same year, Li et al. also published a study examining the adsorption of three different pesticides (imidacloprid, buprofezin, and difenoconazole) onto the surface of polyethylene microplastics.^76^ Their methodology was quite similar to that used by Chen et al., but a key difference involved the use of Grand Canonical Monte Carlo (GCMC) in addition to MD simulations. GCMC is distinctly different from MD; instead of evolving a system through time, random modifications are made (e.g., the addition or removal of pollutant molecules from the simulation box), which allows the user to understand if a pollutant can be absorbed inside of a polymeric system (as opposed to external adsorption). Their simulations used the COMPASS II force field, a simulation time of 200 ps (1 fs timestep), a polymer with DoP = 160, and a temperature of 298 K in the NVT ensemble. Their results showed that none of the pesticides were able to absorb into the free space of the model FPP (the free space calculated was to be 4752 Å^3^). This result aligns with existing knowledge that microplastics can adsorb pollutants onto their surface, but there is limited evidence to suggest that FPPs can absorb pollutants into their interior.^67^ Their MD simulations showed that buprofezin had the strongest interaction with polyethylene (−25.0 kcal/mol), followed by difenoconazole (−22.4 kcal/mol) and imidacloprid (−20.2 kcal/mol). In batch adsorption experiments, polyethylene had a significantly higher adsorption capacity for difenoconazole than for both buprofezin and imidacloprid, which had adsorption capacities similar to one another. The observed trend in the MD-calculated interaction energies did not strongly align with the trend observed experimentally; however, with only three data points, no large-scale inferences can be made, and naturally due to the limited data set, there are difficulties in understanding the full potential of MD simulations in this setting.

In 2023, a few notable publications used MD to study FPP adsorption processes. For example, Leng et al. examined the adsorption of 17β-estradiol onto the surface of polyethylene, polypropylene, and polystyrene microplastics.^77^ 17β-estradiol is a natural steroidal estrogen and a known endocrine-disrupting chemical found in aquatic environments; therefore, with the increasing prevalence of FPPs in the environment, it is important to acquire a molecular-scale understanding of how it adsorbs.^78^ Similar to the previously discussed approaches from Wang,^69,79^ Chen,^71^ and Li,^76^ batch adsorption methods were utilized, and isotherms were produced to examine the adsorption process. In their MD simulations, Leng et al. used the more recently developed COMPASS II force field in Materials Studio, which is likely to provide better performance than the force fields used in some of the earlier studies.^72^ Their model microplastics each had a different number of repeating units (RU) in the polymer chains (PE = 300 RU, PP = 250 RU, and PS = 100 RU). Their simulation timescale was chosen to be 200 ps, and they used a temperature of 298 K, which was controlled by using the Nosé–Hoover thermostat.^80^ The results showed that PE had the highest adsorption capacity (0.642 mg/g), followed by PP (0.545 mg/g), and PS (0.415 mg/g). Importantly, the MD-calculated interaction energies were again in concordance with the experimentally calculated adsorption capacities: PE (−26.06 kcal/mol) had a stronger interaction than both PP (−25.19 kcal/mol) and PS (−23.79 kcal/mol). This study provides another example of MD simulations being able to predict the relative adsorption capacities for a range of different plastic polymers. Also in 2023, Dias et al. examined the adsorption of the pesticide atrazine (ATZ) and two hormones (testosterone and progesterone, TTR and PGT, respectively) onto the surface of polyamide microplastics.^81^ In a very similar approach to Chen et al., in what is clearly a trend in the literature, batch adsorption experiments, sorption isotherms, and MD were combined to study the adsorption process. However, in addition to MD, density functional theory (DFT) calculations were also performed. Their batch adsorption experiments involved mixing 20 mg of PA microplastics and 2 mL of contaminant together in a glass vial at room temperature. The contaminant concentrations were fixed, and over a period of 108 h, the liquid contaminant concentrations were assessed using liquid chromatography-tandem mass spectrometry (LC-MS/MS) at various time points. Their isotherm experiments were carried out using concentrations ranging from 0.01 to 2.0 mg/L, and all experiments were performed in triplicate. Their MD simulations each involved a single pollutant molecule and a small polyamide (4 RU, C_26_H_50_N_4_O_4_) being placed into a cubic box of length 30.5 Å. The OPLS-AA force field was used throughout the simulations, PBCs were utilized in each dimension, and the simulation length was 30 ns (resulting in 30 000 unique configurations). To calculate a representative adsorption energy across the whole simulation, a single MD configuration was taken for every 938 configurations, resulting in 32 unique configurations being taken forward for DFT calculations. For each of the 32 configurations, the single-point energy was calculated with DFT at the M06-2X/cc-pVDZ level of theory (in both the gas phase and with the SMD solvent model for water), and the adsorption energy was calculated according to
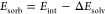
9where *E*_sorb_ is the adsorption energy, Δ*E*_solv_ is the solvation energy, and *E*_int_ is the interaction energy calculated according to eq 8. This is a particularly interesting approach compared to the previously discussed studies; the solvation energy was calculated by
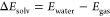
10where *E*_water_ is the average DFT single-point energy across the 32 configurations in the SMD solvent model, and *E*_gas_ is the average DFT single-point energy across the 32 configurations in the gas phase. By the inclusion of a term such as Δ*E*_solv_, the energy cost associated with the pollutant moving from the bulk water to the plastic surface is accounted for. It is worth noting, however, that implicit solvation does not always provide accurate results in comparison with explicit solvation, but SMD has shown to be a good implicit solvent model for solvation energies.^82^ Further, it is unclear if Dias et al. utilized absolute electronic energies or the thermally corrected free energies calculated with DFT;^83^ thermal corrections should typically always be included and could be further improved by using a quasi-harmonic approximation to the free energy.^84^ Experimentally, their results showed that each pollutant was capable of adsorbing onto the surface of polyamide microplastics, and the Langmuir isotherm provided the best fit to their data, indicating that electrostatic and van de Waals dominated monolayer coverage was likely occurring.^85^ Exact adsorption capacities in terms of concentration were not provided, but adsorption efficiencies were calculated in terms of percentages, and it was shown that PGT had the highest efficiency (∼90%), followed by TTR (55–80%) and ATZ (∼20%). Results of their simulations showed that PGT had the lowest *E*_sorb_ value (12–15 kcal/mol) and therefore had the highest tendency to adsorb to the surface of the polymer. This was followed by TTR (13–16 kcal/mol) and ATZ (19–22 kcal/mol), which is in direct agreement with the adsorption efficiencies calculated experimentally. Additionally, the simulations revealed that noncovalent interactions such as hydrogen bonds and van der Waals interactions were dominant in the adsorption process, supporting the results obtained from the isotherm experiments. This aspect of MD simulations can be particularly useful; it can elucidate (i) the type of interactions present (e.g., H-bonds) and (ii) the atoms involved in the process, permitting a highly detailed molecular-scale understanding of interfacial processes.

Liu et al. published a unique, holistic approach to study the adsorption of aromatic hydrocarbons in both freshwater and artificial seawater onto the surface of FPPs.^86^ They combined batch adsorption experiments, MD, and DFT calculations to study the adsorption of benzene and naphthalene onto the surface of PE and PS. In their MD simulations, similar to previously published studies, a different degree of polymerization was chosen for both PE (50 RU) and PS (100 RU). However, their simulations also included an explicit solvent, whereby approximately 32 000 water molecules were placed into the simulation box. Explicit solvation has not been considered in most of the existing literature, and studies would only benefit from this inclusion. Additionally, for the seawater system, NaCl was also included in the simulation box to account for the effect of salinity. They used GROMACS and the OPLS-AA force field to perform the simulations and chose a temperature of 300 K with the Parrinello–Rahman thermostat.^87^ Both the NPT and NVT ensembles were used for equilibration and production runs. In addition to MD simulations, Liu et al. also utilized a quantum chemical approach (DFT) to study the adsorption process. Due to the computational expense of DFT, polymer chains including 50+ RU are typically untenable at this level of theory; therefore, a much smaller system was considered. This included a polyethylene of 3 RU, a single aromatic hydrocarbon, and three water molecules. The interaction energy was calculated similarly to eq 8, but it instead included a term to account for basis superposition error.^88^ Results of their batch adsorption experiments showed that the microplastic adsorption capacities were always enhanced in artificial seawater, supporting previous accounts of increased salinity leading to decreased solubility of organic pollutants, resulting in enhanced adsorption to microplastic surfaces.^89^ To support this, their MD results also showed that adsorption was improved in model seawater; the MD-calculated interaction energies were always more negative in model seawater compared to those in model freshwater. For example, in the polyethylene–benzene system, the interaction energy in pure water was −9.07 kcal/mol compared to −28.71 kcal/mol in model seawater. Interestingly, the results of their MD simulations showed that benzene and naphthalene were capable of absorbing into the model microplastic pores. Upon entering the pores, the hydrocarbons were shown to modify the polymer structure, resulting in a stabilization of their confinement to the pore regions. Solvent accessible surface area (SASA) calculations were also carried out to calculate the surface areas of the polymer chains that were accessible to a solvent.^90^ The results showed that the SASA of their model FPPs was increased in model seawater, indicating that a larger surface area was available for sorption compared with the pure freshwater systems. The combination of batch adsorption experiments with MD simulations provides evidence that FPP adsorption processes are enhanced in saltwater environments such as oceans and estuaries.^12,91^ Additionally, by adopting these two approaches in conjunction with one another, both a macroscopic- and molecular-scale understanding of the process are acquired, along with greater confidence in both conclusions.

**Figure 5:**
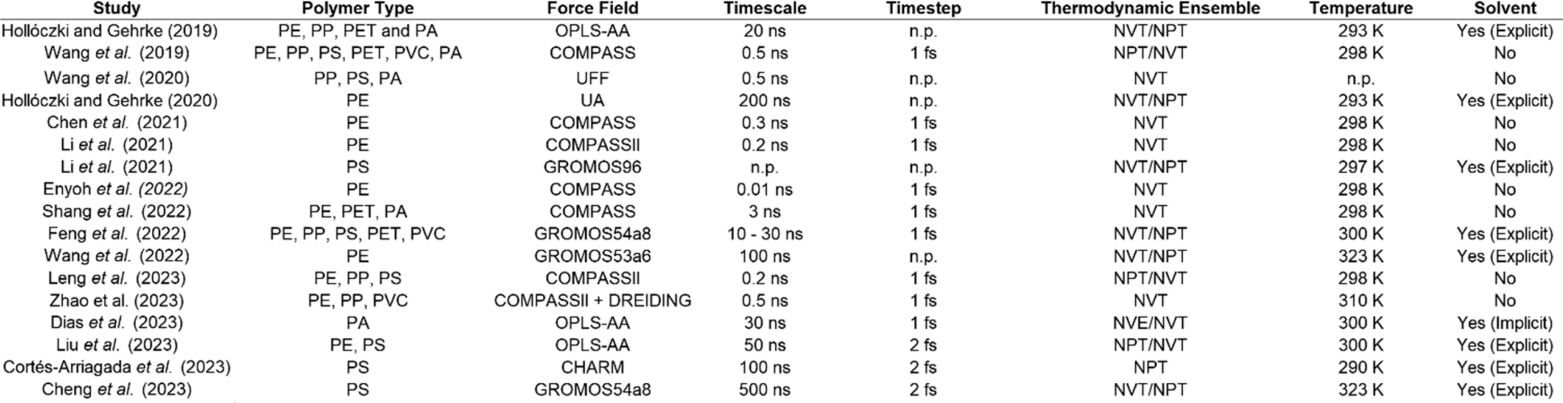
A summary of the polymer types, force fields, timescales, timesteps, thermodynamic ensembles, temperatures, and solvents for the main studies reviewed in this work. “n.p.” indicates that this information was not provided by the authors.

In contrast to the experimental–theoretical approaches discussed above, there are some studies that adopt a wholly computational approach to studying adsorption processes. Along with other commonly utilized PPCPs and pesticides, PFAS (per- and polyfluoroalkyl substance) compounds are widely manufactured and commonly used substances in modern society, and they are of great concern as emerging contaminants due to a range of known and unknown health effects.^92^ In 2022, Enyoh et al. studied the adsorption of seven PFAS compounds onto the surface of a model microplastic of polyethylene.^93^ Enyoh et al. chose PFAS substances based on their commonality in society: these were perfluorononanoic acid (PFNA), perfluorohexanesulfonic acid (PFHxS), perfluorohexanoic acid (PFHxA), perfluorodecanoic acid (PFDA), perfluorooctanesulfonic acid (PFOS), perfluorobutanesulfonic acid (PFBS), and perfluorooctanoic acid (PFOA). They used a combination of MD and GCMC simulations and did not supplement their simulation results with an experimental approach. Their MD simulations utilized the COMPASS force field and a temperature of 298 K, and the temperature was controlled by the Berendsen thermostat in the NVT ensemble. Their results showed that all PFAS compounds were capable of adsorbing onto the surface of the model microplastic and that the magnitudes of the interaction energies (ranging from −103.4 to −712.9 kcal/mol) were much greater compared to those from previous studies.^69,76^ This key result indicates that PFAS compounds are likely to adsorb onto the surface of FPPs, with the MD simulations providing supporting evidence in alignment with previously published experimental results.^94^ The adsorption process at the FPP interface has also been considered for less commonly encountered material types. For example, covalent organic frameworks (COFs) are a group of porous materials that can be utilized as adsorbents, and they show a range of desirable properties such as wide-ranging chemical functionality, easily tunable structures, and high chemical stabilities.^95^ Compared to more conventional adsorbents, many of which having pore sizes greater than nanoplastic polymers, COFs could potentially be used as nanoplastic adsorbents due to their smaller pore sizes. Thus, the use of COFs for environmental remediation must be explored. Shang et al. published some detailed work examining the adsorption of nanoplastics (PET, PE, and PA) in a range of different COFs.^96^ Their simulations were carried out with TpPa-H (and other closely related functionalized derivatives, e.g., TpPa-CH_3_) and three different nanoplastic polymers, each containing a similar number of atoms (PE: 28 RU, C_55_H_114_; PET: 8 RU, C_80_H_66_O_32_; and PA, 9 RU, C_54_H_101_N_9_O_9_). The COMPASS force field was used throughout, and all equilibration runs were carried out for 1 ns. Production runs were carried out for an additional 3 ns using the Verlet integration method in the NVT ensemble. The average interaction energies were calculated, and it was shown that polyethylene showed the weakest adsorption to TpPa-H (−70.45 kcal/mol), followed by PA (−78.69 kcal/mol) and PET (−97.97 kcal/mol), which showed the strongest adsorption. Additionally, it was shown that PET showed the greatest penetration of the channels in TpPa-H, likely leading to stronger intermolecular interactions and improved adsorption. The effect of functionalization was then investigated with the strongest adsorbing polymer (PET), and it was shown that bulky groups (e.g., TpPa-CH_3_) led to decreased adsorption, while polar functionalization (TpPa-OH, TpPa-NO_2_, and TpPa-F) led to increased adsorption. Their MD simulations also showed that van der Waals forces were the dominant forces in all simulations, that increased electrostatic interactions were present in certain cases (due to the presence of polar groups in PET and PA), and that increased penetration by the polymer was the main contributing factor to increased adsorption and lower interaction energies. Owing to the need for understanding the interaction mechanisms of numerous different organic pollutants at microplastic surfaces, Cortés-Arriagada et al. published another computational approach, wherein they examined the adsorption of seven commonly used PPCPs onto the surface of polystyrene.^97^ Similar to Liu et al., they used a combination of force field methods (such as MD) and quantum chemical methods to study the mechanism of adsorption.^86^ However, in this approach, they utilized energy decomposition analysis (EDA), wherein the interaction energies are decomposed into numerous physical contributors:^98^
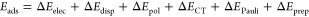
11where *E*_ads_ is the adsorption energy, Δ*E*_elec_ is an electrostatic term, Δ*E*_disp_ represents the dispersion forces that arise due to van de Waals interactions, Δ*E*_pol_ is a term that accounts for polarization, and Δ*E*_CT_ is a term that accounts for charge transfer.^99^ Δ*E*_Pauli_ and Δ*E*_prep_ are terms that increase the overall energy and correspond to energy destabilization due to Pauli repulsion (Δ*E*_Pauli_) and an energy penalty that arises due to geometric differences from the isolated polymer (Δ*E*_prep_). Naturally, understanding the nature of the intermolecular interactions between a model microplastic and a potential pollutant will aid in developing a detailed picture of the adsorption process that occurs prior to the transport and release of pollutants. Thus, they constructed a model microplastic consisting of 338 atoms (C_168_H_170_), used the CHARMM force field, and ran simulations at a target temperature of 290 K (a close approximation of the average temperature of seawater). An explicit solvent was also utilized (the TIP3P model for water) throughout some of their simulations, but separately, implicit solvation (the SMD model) was also included to permit the use of ALMO-EDA, a mathematical framework that permits EDA following the application of an implicit continuum solvent model.^100,101^ Interaction energies were calculated using DFT, and similar to previous studies, the adsorption process was favorable with all interaction energies having negative values. The details of the whole in silico approach adopted by Cortés-Arriagada et al. are significant and beyond the scope of this Review, and therefore, only the MD results will be discussed. From their MD data, they examined the thermodynamic stability of the microplastic–pollutant systems, and three values were of particular interest in this study: the radius of gyration (RG), the root-mean-square deviation (RMSD), and the center of mass (COM). In the context of polymers, the RG allows the user to understand the effective size of the system; for example, a small RG value is indicative of a compact polymer that spends most of the simulation in a folded form. For all seven PS models, a compact structure was obtained with RG values sitting between 7.5 and 8.2 Å. These values provide valuable insight into how the structure of the polymer changes upon exposure to the pollutant, and it is clear from their results that only minor structural changes were observed following adsorption. The COM describes the geometrical distance between the PS center of mass and each PPCP, allowing the user to acquire a molecular-scale picture of the changes in the distance between the polymer and pollutant; that is, the adsorption and desorption of the pollutant from the polymer surface. Finally, the RMSD is a similarity measure between the simulation starting point and a particular timestep in the future. Their results showed a wide range of COM and RMSD values across all PPCP–PS systems, but importantly, this study demonstrates the strength of using these values together to study the adsorption process. For example, Cortés-Arriagada et al. noted that for the naproxen–PS system, a stable COM was observed throughout most of the simulation, with an average distance between naproxen and the model microplastic of 1.4 Å. After 80 ns, the COM value increased to around 30 Å before dropping close to its initial value. When the COM value increased, a simultaneous increase was seen in the RMSD, but following a reduction in the COM value, the RMSD continued to increase. Although the significance of this result might not be immediately apparent, this is direct evidence that supports the movement of the pollutant from one adsorption site to another. In addition to this, the EDA results demonstrated that hydrogen bonding, π-type interactions, and specific electrostatic interactions (e.g., C–H) were the main stabilizing sources. To the best of the author’s knowledge and at the time of this publication, Cortés-Arriagada et al. have produced the most in-depth study that uses MD to study microplastic adsorption, and future studies should take particular note of this publication (see Figure 5).

### 3.2 Adsorption of Inorganic Pollutants to FPPs

**Figure 6:**
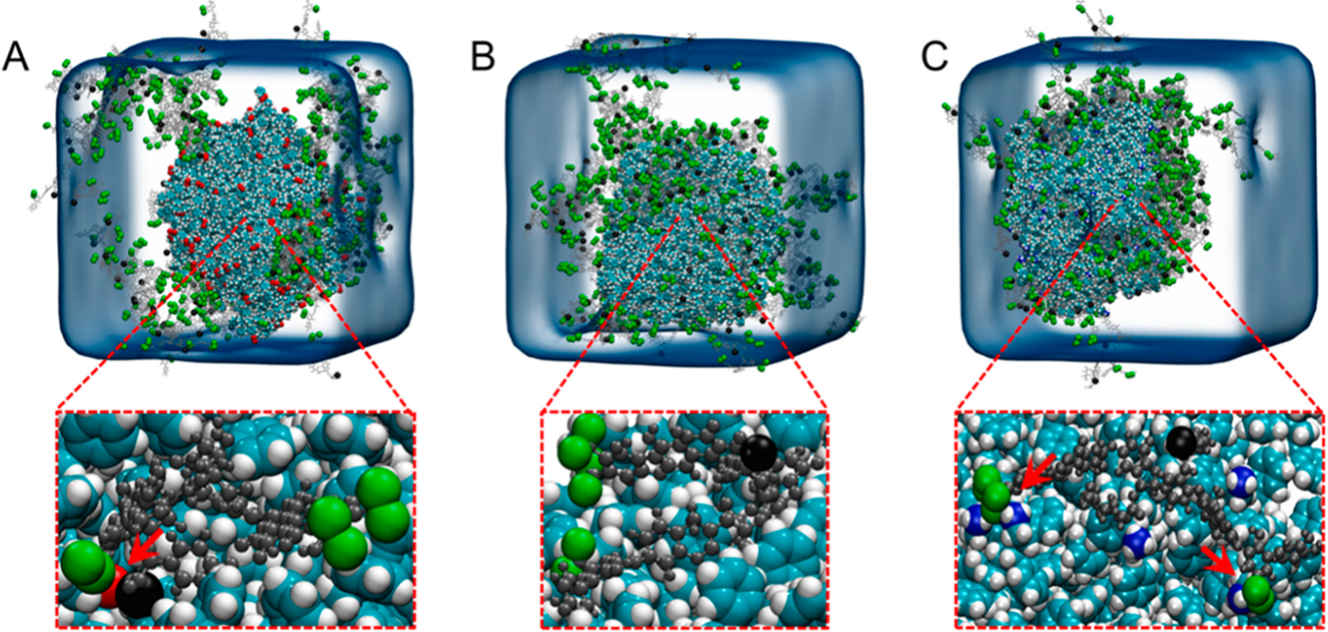
Adsorption of humic acid onto model polystyrene nanoplastics with different surface charges: (A) anionic, (B) neutral, and (C) cationic. Reprinted in part with permission from ref (102). Copyright 2022 Elsevier.

Although organic pollutants are a major concern, the adsorption of inorganic pollutants also poses a significant risk. Wang et al. studied the adsorption of Sr^2+^, a known aquatic pollutant found in close proximity to nuclear power plants, onto PA, PS, and PP polymer chains.^79^ Each batch experiment was carried out with 45 mg of microplastics, MD simulations were carried out in Materials Studio, and the Universal Force Field (UFF) was used for energy calculations. They conducted adsorption isotherm experiments in combination with MD simulations, and their results showed that the maximum adsorption capacities for Sr^2+^ were 31.8, 51.4, and 52.4 μg/g for PA, PS, and PP respectively, with the nonlinear Temkin model being the optimal isotherm. Their MD-calculated interaction energies aligned with the experimental adsorption capacities, although it must be noted that the interaction energy difference seen between PA and PS was 0.8 kcal/mol, while their sorption capacities differed by 19.6 μg/g. However, in the case of PS and PP, their interaction energies differed by 6.64 kcal/mol, while their sorption capacities showed a much smaller difference of only 1 μg/g. This could relate to issues with the chosen force field; energy calculations (and thus the chosen force field) have a significant effect on the simulation accuracy, and UFF has been previously shown to have unreliable results in conformational analysis.^42^ This study indicates that although a trend was observed between the Sr^2+^ experimental isotherms and MD simulations, the magnitudes were not captured by their chosen computational approach. Additionally, SrCl_2_ was used in their MD simulations, which is explicitly different from the solvated Sr^2+^ ion that would be present experimentally. Feng et al. published an exciting approach wherein the adsorption of humic acid, benzo[*a*]pyrene, and Cu^2+^ was considered on the surface of PE, PP, PS, PET, and PVC model nanoplastics.^102^ It has previously been shown that dissolved organic matter (DOM), such as humic acids (HAs), can form an eco-corona on the surface of FPPs, modifying their surface and behavior in aquatic environments. By forming an eco-corona, DOM can therefore regulate and modulate the adsorption of pollutants at FPP interfaces. Not only can DOM itself interact with pollutants but, once it is adsorbed onto the surface of FPPs, it can further enhance adsorption due to the wide-ranging chemical functionality present in its structure (e.g., charged carboxyl groups, charged amine groups and aromatic rings; see Figure 6). In this approach, five polymers were constructed in Materials Studio 2017, each with the same degree of polymerization (each consisting of 20 RU). Models for both polymeric particles and plastic films (surfaces) were generated to permit a direct comparison between different geometric structures. Although HAs can be wide-ranging in terms of their chemical structure, Feng et al. used the Stevenson model for HAs, consisting of 158 atoms, 2 charged carboxyl groups, and a single charged amine group. Benzo[*a*]pyrene (BaP) was included as a prototypical polyaromatic hydrocarbon, and Cu^2+^ was included as a prototypical heavy metal. To prepare the simulation, 140 HA molecules, 60 BaPs, 100 Cu^2+^ ions, and 99 344 water molecules were added to a simulation box that was 15 nm^3^ in dimension. Plastic surfaces were prepared by adding 64 polymer chains to a simulation box (10 nm × 10 nm × 60 nm), followed by a simulation in the NVT ensemble at a high enough temperature to reach a high mobility melting state. Following annealing (changing the temperature at a rate of 0.1 K/ps), each polymeric system was explicitly solvated with water, and a 10 ns simulation was carried out in the NPT ensemble (300 K). BaP was then adsorbed onto the surface of each plastic, and the polymer with the highest adsorption capacity (PS) was taken forward for additional simulations with the HA–pollutant mixture. All simulations were carried out in GROMACS 4.6.7 with the GROMOS 54a8 force field, and the temperature was maintained with the V-rescale thermostat.^103^ A 1 fs timestep was used in all simulations, and PBCs were applied in all directions. Their results showed that PS had the highest adsorption capacity for BaP and that plastic surfaces showed stronger adsorption than individual polymers (BaP was adsorbed on both plastic surfaces and individual polymers). PS had the lowest interaction energy by a wide margin (about −140 kJ/mol), and the molecular mechanism of adsorption was elucidated. It was shown that BaP became anchored into the PS film and then intercalated between two aromatic rings, resulting in π–π stacking interactions. Results from the HA–pollutant simulations showed that BaPs became encapsulated inside the hydrophobic region of the HA assembly and made greatest contact with internally located aromatic carbons. In contrast, Cu^2+^ ions were shown to bind mostly to negatively charged carboxyl groups that were unbound from the intramolecular positively charged amine groups. Their final simulations included a model nanoplastic, HAs, BaPs, and Cu^2+^ ions, and the results showed that eco-coronas were capable of forming at the nanoplastic interface. Additionally, HAs were capable of competitively binding BaPs, meaning that less direct adsorption took place on the nanoplastic surface. This study and its results are noteworthy for a few reasons: not only are adsorption processes being probed broadly at the molecular scale but cooperative and competitive binding mechanisms are being examined in a multicomponent system. Although this still represents a simplified system relative to what might be found in nature, it takes a significant step forward in thinking about the known chemical complexity found in natural waters where FPPs are found in abundance.

### 3.3 Biomolecular Interactions at the Interface of FPPs

Although it is clear that MD plays an exciting role in understanding the adsorption process, numerous studies have used MD to examine the interaction between FPPs and other systems of biological interest. To provide insight into some of the pioneering studies and to provide an overview of some other exciting applications of MD simulations in the field of plastic pollution, some of these studies are highlighted below.

**Figure 7:**
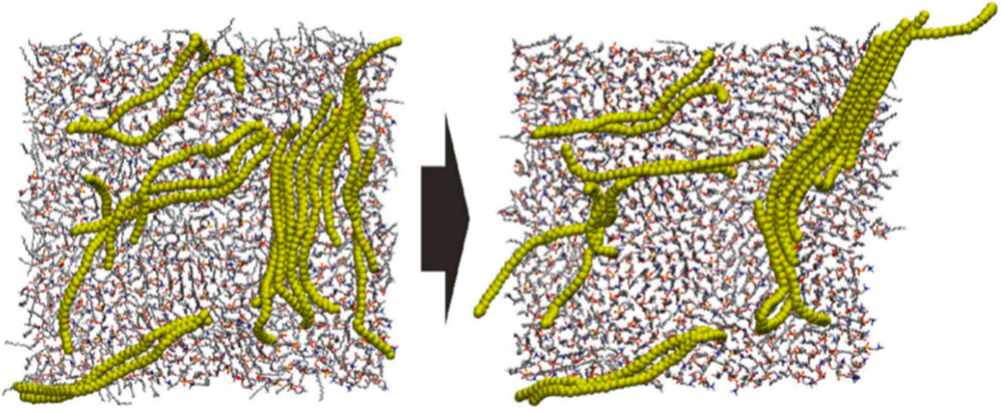
Disentangled PE chains were seen in a POPC membrane after 200 ns of simulation time. This led to an increase in the polymer surface area throughout the course of the simulation. Reproduced from ref (104) - CC BY 4.0 (http://creativecommons.org/licenses/by/4.0/).

To the best of the author’s knowledge, the first example of MD being used explicitly in the context of FPP adsorption came from Hollóczki and Gehrke in 2019.^16^ In this seminal work, they used MD to examine the effect of single polymer chains (NPs of PE, PP, PET, and nylon-6,6) on two of the most prevalent types of secondary structures found in proteins. They constructed two peptide models: a tryptophan zipper to account for a β-sheet and a polyalanine α-helix containing 12 alanine molecules. Their simulations were carried out in LAMPSS, and the OPLS-AA force field was used to model the polymers, amino acids, and peptides. In each simulation, solvation effects were included through the inclusion of 10 000 water molecules (the SPC/E water model), permitting the effects of an external aqueous environment to be explicitly accounted for. The results showed that both peptide structures adsorbed predominantly on the hydrophobic regions of the NPs, and in the case of the nylon polymer, the polyalanine α-helix spontaneously changed into a β-looplike structure at the surface of the particle. This result has significant implications and explicitly demonstrates that NPs can modify the secondary structure of proteins. Additionally, this is the first example of MD being used to study adsorption in a large multicomponent system containing an FPP. This study played a key role in the formulation of future studies in which adsorption was considered in the context of different FPPs and pollutants. A year later, the same authors published another seminal proof-of-concept study on how nanoplastics could interact with cell membranes.^104^ Due to the key role of the cell membrane in biology (e.g., cell signaling and barrier to entry), a detailed understanding of how nanoplastics could interact with, disrupt, or damage membrane bilayers must be developed. In this work, Hollóczki and Gehrke simulated a globular PE nanoplastic (5 nm in diameter) as a transmembrane object in a phosphatidylcholine (POPC) bilayer. Simulations were carried out in the LAMPSS package with the united atom force field. Equilibration runs were carried out for 5 ns, followed by 200 ns production runs at 293 K and 1 bar of pressure. Their results showed that the cell membrane enabled the nanoplastic to be broken down into smaller polymer chains and that the surface area of the nanoplastic almost doubled over the course of the simulation (see Figure 7). Additionally, it was shown that the presence of the nanoplastic altered the overall structure of the membrane, particularly with respect to the internal lipid side chains. In concert, the two studies published by Hollóczki and Gehrke played a pivotal role in paving the way for other studies to examine the effect of FPPs on biomolecular systems. A couple of years later, Wang et al. used cell culture experiments and MD to examine the effect of PE microplastics on cell membrane integrity.^105^ They exposed HepG2 cells to differing concentrations of microplastics for 24 h before visualizing the cells using a fluorescence microscope. MD simulations were performed wherein four different systems were created: a no-load system that contained only a dipalmitoylphosphatidylcholine (DPPC) cell membrane, a system containing one PE10 polymer and DPPC, a system containing 8 PE10 polymers and DPPC, and finally, a system containing 2 PE10, 2 PE20, 2 PE40, and 2 PE60 polymers (designed to represent a multicomponent mixture of different polymers with differing degrees of polymerization). Polymer chains were randomly placed into an aqueous environment (SPC model for water) close to the upper membrane of DPPC, and all final simulation runs were carried out for 100 ns with the GROMOS 53a6 force field. Their results showed that within 50 ns, all PE polymers spontaneously transferred from the external aqueous phase to the hydrophobic internal region of the DPPC bilayer, indicating that small nanoplastic polymers can easily enter cell membranes and remain stable. This study provides a good example of MD being used in the field of biomolecular simulation and provides a powerful molecular-scale picture of how FPPs can interact with the outside of cells in an aqueous environment. Chen et al. published an interesting approach wherein full factorial design was used to predict the combined toxicity of different plastic components toward the zebrafish (*Danio rerio*, a commonly employed model organism).^106^ The degree of toxicity was defined as the total binding energy of all plastic components in the cytochrome P450 receptor. Their factorial design included six factors (A, styrene monomer; B, plasticizer; C, antioxidant; D, flame retardant; E, light stabilizer; and F, heat stabilizer), which account for a range of plastic additives that can be used to improve the material properties. Two levels were included in the factorial design (0—low level, 1—high level), whereby each level describes a different chemical compound within the same group as the factor (e.g., A-0 is a single monomer of styrene, while A-1 is a polystyrene consisting of 5 RU, or C-0 is nonylphenol, and C-1 is acetone diphenylamine, both of which are antioxidants). Each combination from the full factorial design process was docked into the P450 receptor, and the toxicity was quantified by the magnitude of the binding energy; lower binding energies correspond to more stable systems and therefore a greater toxic effect. Their MD simulations were carried out in GROMACS, and the molecular mechanics Poisson–Boltzmann (MM-PBSA) method was used to calculate the binding energies; MM-PBSA is a method employed to integrate high-throughput MD simulations with binding free energy calculations.^107^ The multiligand–receptor complex was solvated by using the SPC216 model in GROMACS, followed by the addition of sodium ions to create an electrically neutral system. Simulations were carried out at 297 K with both the NPT and NVT ensembles at different times. In total, 64 binding energies were calculated, and numerous interesting observations were seen. For example, it was shown that when all other additives were kept constant and only the degree of polymerization of polystyrene was changed (e.g., a single monomer and 5 RU), the binding energy was significantly lower (−232.2 kJ/mol) for the single monomer as opposed to the polymer (−65.6 kJ/mol) consisting of multiple RU. This result could infer that greater decomposition of polystyrene in the marine environment could cause a greater toxic effect in combination with other plastic additives. Their approach also showed that particular components were common across the multiligand–receptor complexes, e.g., those with the lowest and highest overall binding energies, providing insight into the risk of toxicity for individual additives present in a mixture. This approach was notably novel compared to other publications in the area and provides an interesting template by which to study the toxicity of chemical mixtures. However, there are some factors to consider in their approach; for example, does a lower binding energy always infer that the toxicity of a particular compound will be greater? Given the role of P450 enzymes (e.g., metabolism of xenobiotics), it is possible that lower binding energies could equate to reduced toxicity due to faster metabolism.^108^ Further, it is possible that the interaction with P450 could result in more toxic metabolites being produced, which is not accounted for in their in silico approach.^109^ Despite this, a full factorial design could provide a unique method by which MD and molecular docking could be used to improve the universal understanding of the chemical features and intermolecular interactions responsible for toxicity upon exposure to FPPs.

Most recently, Zhao et al. published a combined experimental–computational approach wherein the adsorption of three nanoplastic polymers onto the surface of eight different *Lactobacillus* strains was examined.^110^ These bacterial strains are known probiotics and could act as potential adsorbents to remove nanoplastics from food. Therefore, understanding the potential for adsorption at the interface between an NP and a bacterial cell wall could prove to be of great value. NP adsorption was also examined on different bacterial cell components following their separation; peptidoglycan (PG), teichoic acid, exopolysaccharides, and surface-layer proteins were all separated. Their results showed that all strains were capable of adsorbing nanoplastics (% nanoplastic adsorbed ranged from ∼25% to 72%) and that PG (% adsorbed: 51.8% for PE, 55.7% for PP, and 59.3% for PVC) had a much higher adsorption capacity than the other cellular components. Thus, in line with this result, they carried out MD simulations using Materials Studio 7 and constructed a model peptidoglycan cell wall from the ZP-6 *Lactobacillus* strain. All simulations were carried out under the NPT ensemble at 310 K with the COMPASS II and DREIDING force fields. The simulation was carried out for quite a short timescale of 0.5 ns with a timestep of 1 fs. The interaction energy was calculated similarly to eq 8, whereby the energy was calculated for the PG–NP system and the isolated PG and nanoplastic systems. Their interaction energies ((PE) −115.8 > (PP) −134.4 > (PVC) −159.5 kcal/mol) showed the same trend observed in the experimental adsorption capacities, with all NPs showing stable adsorption; however, PVC showed the strongest overall interaction with the PG wall. This study provides another example of interaction energies providing alignment with the experimentally determined adsorption capacities, but it is yet again limited by the number of molecules included in the study. Another recent study by Cheng et al. considered adsorbed BaP on polystyrene NPs and how adsorption may affect NP movement through a phospholipid cell membrane.^111^ Their simulations were carried out in GROMACS, and they also used the GROMOS 54a8 force field, along with very similar simulation parameters to the approach taken by Feng and co-workers.^102^ Their results showed that BaP enhanced and promoted the permeation of NPs through the cell membrane, resulting in a depolymerized polymer within the cell membrane. In addition, the membrane fluidity notably decreased, which could potentially lead to enhanced cellular toxicity. Together, these studies show that MD can be used to study a wide variety of biological and nonbiological systems in the context of FPPs, and it is clear that these approaches for simulating multicomponent systems present a very exciting future for the study of interfacial processes in FPPs.

## 4.0 Conclusion

Overall, in this work, the current state of affairs on using MD to study adsorption at the surface of plastic particles was presented. A gentle introduction to molecular dynamics was provided for the unacquainted, followed by considering how previous research has examined the adsorption of (i) organic pollutants, (ii) inorganic pollutants, and (iii) biomolecules at the surface of FPPs. It is clear that the use of MD for studying the adsorption process on FPPs is still in its infancy. However, it is also clear that MD can be used to great effect in this area; not only can the underlying molecular mechanisms of adsorption be elucidated but novel insight can be gained into the large-scale structural changes that occur due to the presence of pollutants and/or biomolecules. From all of the studies presented here, there are some general observations that can be made. First, it is clear that no single force field is dominant in terms of choice. COMPASS and its variants are the most well-represented, while the GROMOS force fields are also a popular choice. The UFF and united atom (UA) force field are each represented only by a single study, and it is clear that no general consensus has been reached on which force fields provide the best insight into the adsorption process. Therefore, the literature would benefit from benchmarking studies wherein different force fields are examined for their performance toward a single model system. Similarly, many of these studies make little or no reference as to why a particular force field was chosen; without making an informed choice, the resultant data could be notably weak if not totally incorrect. It is also clear that up until this point, numerous studies performed their simulations over a very short timescale. From a modern perspective on the field, simulations that run for tens of nanoseconds can still be considered notably short, and some of the studies presented here operated for less than a single nanosecond.^55^ Given that microsecond timescales are now achievable in protein simulations, this is a factor that must be more carefully considered for future studies in this area.^112^ It is good to see that a wide variety of polymer types have been considered, but in future studies, it would be advantageous to see larger-scale data sets that compare interaction energies with adsorption capacities. Although many studies show encouraging results where MD and experimental data are in direct alignment, much larger data sets are required to derive larger-scale inferences. Larger-scale data sets could potentially revolutionize the initial assessment of pollutants and their interaction with FPPs, enabling better prioritization of chemical pollutants. Could we arrive in a world where MD could be used as a direct replacement (in many instances) for time-consuming experimental methods that only contribute more, albeit at a small scale, to global pollution.

## Abbreviations

DFT: Density functional theory
FPP: Fine plastic particle
MD: Molecular dynamics
MP: Microplastic
NP: Nanoplastic
